# Ruptured Aneurysm of the Splenic Artery: A Rare Cause of Abdominal Pain after Blunt Trauma

**DOI:** 10.5812/traumamon.8271

**Published:** 2013-05-26

**Authors:** Jalalludin Khoshnevis, Saran Lotfollahzadeh, Mohammad Reza Sobhiyeh, Hossein Najd Sepas, Masomah Abbas Nejad, Ali Rahbari, Nazanin Behnaz, Zeinab Mahdi

**Affiliations:** 1 Department of Vascular Surgery, Shahid Beheshti University of Medical Sciences and Health Services, Shohadaye Tajrish Hospital, Tehran, IR Iran; 2 Department of Pathology, Shahid Beheshti University of Medical Sciences, Tehran, IR Iran

**Keywords:** Splenic Artery, Aneurysm, Abdominal Pain, Male

## Abstract

**Introduction:**

Splenic artery aneurysms (SAAs) are rare (0.2-10.4%); however, they are the most common form of visceral artery aneurysms. Splenic artery aneurysms are important to identify, because up to 25% of the cases are complicated by rupture. Post- rupture mortality rate is 25% -70% based on the underlying cause. Herein we present a young patient with abdominal pain after blunt abdominal trauma due to rupture of an SAA.

**Case Presentation:**

A 27-year-old male, without a remarkable medical history, who suffered from abdominal pain for 2 days after falling was admitted to the emergency department with hypovolemic shock. Upon performing emergency laparotomy a ruptured splenic artery aneurysm was found.

**Conclusions:**

It is important to consider rupture of a splenic artery aneurysm in patients with abdominal pain and hypovolemic shock.

## 1. Introduction

Visceral artery aneurysms, albeit rare, are important clinical entities. Rupture of a splenic artery aneurysm (SAA) is accompanied by a high mortality rate of up to 60%(1). SAA is thought to predominantly occur in patients in the sixth decade of life (2, 3) and has been reported in 1% of the general population (4, 5). The incidence of SAA according to gender-grouping revealed a dramatic increase of 75% in multiparous women (6); the fatal rate has been reported to be up to 95% (7). Although the majority of SAA cases are asymptomatic, the signs of SAA rupture can vary from abdominal pain or chest pain, to cardiovascular collapse depending on time the patient reaches the hospital (8). In emergency cases, surgical treatment is the best choice, but in high risk patients and inappropriate candidates for surgery, transcatheter arterial embolization has recently been proven to be a useful alternative in many SAA cases(9).We present a young male patient who referred to the emergency department with abdominal pain after blunt trauma due to the ruptured splenic artery aneurysm.

## 2. Case Presentation

**Figure 1.**
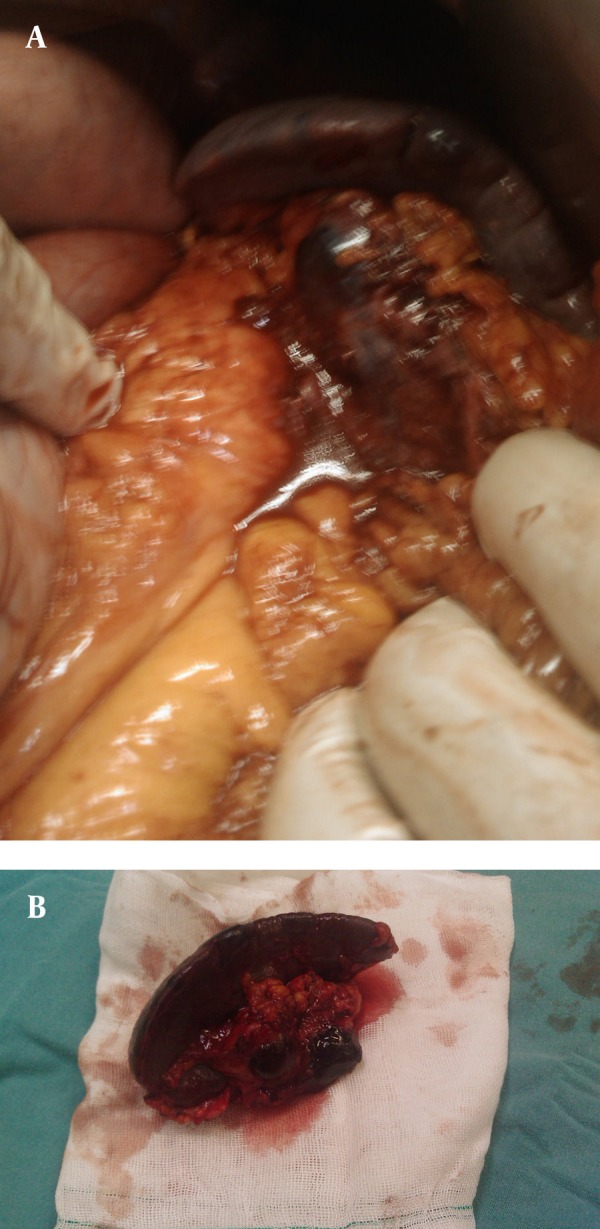
Rupture of Splenic Artery Aneurysm Which Was Located Distally

**Figure 2.**
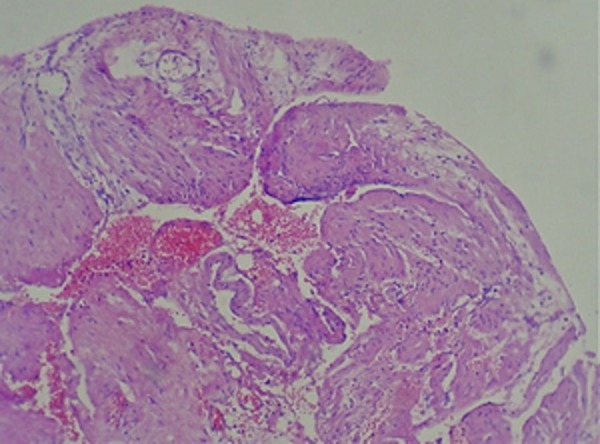
Photomicrograph of Splenic Artery Wall Section Stained with Hematoxylin and Eosin

**Figure 3.**
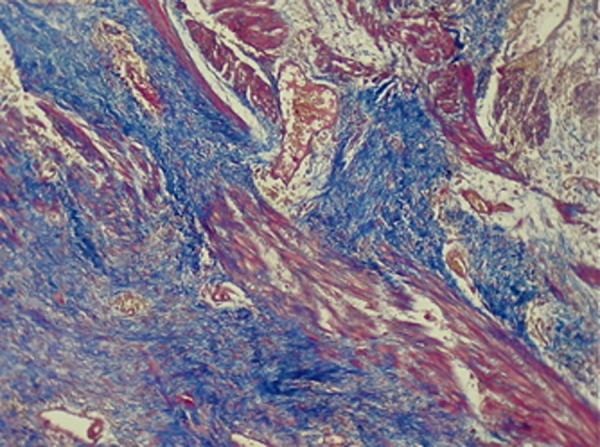
Photomicrograph of Splenic Artery Wall Section Stained with Trichrome Stain

A 27 year-old male referred to the emergency department of our hospital suffering from abdominal pain. His medical history was unremarkable. He was treated symptomatically for 2 days. On admission the patient was conscious. Signs such as dry mouth and pale conjunctiva, were significant. A primary symptom was hypotension. After fluid resuscitation, his pulse rate was 100 beats/min, blood pressure: 100/60 mm/Hg, respiratory rate: 16/min and body temperature: 37°C. His abdomen was not distended, bowel sounds were normal and generalized abdominal tenderness without guarding or rebound tenderness was remarkable. Digital rectal examination (DRE) was normal. Laboratory test results showed mild anemia and leukocytosis with hemoglobin concentration 10g/dl, hematocrit 31%, leukocyte count 14 × 103/µl, platelet count 240× 103/µl. Urine analysis (UA) was normal. FAST (focused abdominal sonography for trauma) revealed free fluid in the abdominal pelvic cavity. After performing FAST, re-evaluation of vital signs were: blood pressure 90/60 mm/Hg, and heart rate 138/min, urine output 30cc/h. Based on haemoperitoneum and the unstable vital signs, the patient underwent exploratory laparotomy; there was 600ccs of blood in the free peritoneal cavity. Moreover, blood accumulation within the peritoneal cavity and retroperitoneal space at the left upper quadrant was detected. The cause of bleeding was rupture of a splenic artery aneurysm located near the hilus (Figure 1 A). Spleen examination was normal, but splenic artery aneurysm was found at the distal part of the splenic artery. Pathological evaluation of proximal ligation followed by aneurysmectomy and splenectomy was performed (Figure 1 B). The postoperative course was monotonous and the patient was discharged on the fifth postoperative day with complete recovery. Histopathological assessment revealed attenuated muscular layer with increased amount of collagen fibers and proliferation of medial vessels with hypertrophic and aneurysmal vascular wall with thrombus (Figures 2 and 3). Congested spleen and unremarkable lymph nodes were also evident.

## 3. Conclusions

Splenic artery aneurysm (SAA) is an uncommon vascular pathology. Usually it is not considered in the differential diagnosis of common abdominal complaints, but it is an important clinical entity because of it may be life-threatening. The most common intra-abdominal true aneurysms may affect infra-renal aorta, the iliac arteries and the splenic artery. SAA is the most common splanchnic artery aneurysm. Most SAA are single, saccular shape and are commonly located in the 1/3 distal part of the splenic artery (3). In our patient, we found a 3 × 2.5 cm saccular aneurysm at the hillus of the splenic artery. Due to advances in new imaging modalities, asymptomatic splenic artery aneurysm is now diagnosed more frequently (10). Therefore about 80 percent of patients with SAA which are asymptomatic are found incidentally (11). Reported incidence of SAA in various autopsy studies ranges from 0.01% to 10.4% in the general population and in high-risk populations respectively (1, 10). The pathogenesis of true SAA is not completely understood, but it may be attributed to blunt abdominal trauma, atherosclerosis, mycotic infection, essential hypertension, portal hypertension, diabetes mellitus, chronic pancreatitis, arterial dysplasia , polyarteritis nodosa, pregnancy or liver transplantation (1, 12). In contrast to true aneurysms of large arteries, atherosclerosis in true SAA is rarely the primary causative factor (13). Our patient was not affected by any of the mentioned underlying pathologies.

SAA is often asymptomatic, and only 20% have symptoms due to rupture causing left upper quadrant or epigastric pain or back pain. In the presented case, the patient was a young male who presented with general abdominal pain and hypovolemic shock, after abdominal blunt trauma which occurred 2 days prior to admission. Clinical presentation of rupture may vary from severe abdominal pain to hypovolemic shock. The majority of aneurysm rupture cases occur in young pregnant women (1). The treatment of true SAA varies from case to case, depending on its locations in the splenic artery. The treatment of distal splenic artery aneurysm has been bipolar surgical ligation of the proximal splenic artery or aneurysmectomy with splenectomy (1). In our case, approximately 600cc blood accumulation within peritoneal cavity and retroperitoneal space at the left upper quadrant was detected. Through retroperitoneal exploration, the source of bleeding was found near distal splenic artery at the splenic hilus. Splenectomy was performed following the ligation of splenic artery proximal to the lesion. Incidental aneurysm can be treated through percutaneous interventions such as transcatheter arterial embolization, or stenting to exclude the aneurysm or percutaneous thrombin injection. The surgeon should keep in mind that despite the rarity of splenic artery aneurysm, splenic artery aneurysm rupture may become a relevant differential diagnosis of intraperitoneal hemorrhage. Though epigastric or flank pain in unstable patients may be a diagnostic clue for the rupture of a visceral aneurysm (1). Regardless of age and gender, in patients with abdominal pain and hypovolemic shock, it is important to consider rupture of splenic artery aneurysm as a possibility.
